# Research on recognition method of leaf diseases of woody fruit plants based on transfer learning

**DOI:** 10.1038/s41598-022-18337-y

**Published:** 2022-09-13

**Authors:** Zhao Wu, Feng Jiang, Rui Cao

**Affiliations:** School of Computer and Information Engineering, Central South University of Forestry and Technology, Changsha, China

**Keywords:** Plant sciences, Electrical and electronic engineering

## Abstract

Fruit leaf diseases have a significant impact on the later development and maturity of fruits, so rapid and accurate identification of fruit leaf diseases plays an important role in the development of fruit production. In this paper, the leaf disease data set of 6 kinds of fruits is divided into 25 categories according to the species—the type of the disease—the severity, and we propose an improved model based on ResNet101 to identify woody fruit plant leaf diseases, in which a global average pooling layer is used to reduce model training parameters, layer normalization, dropout and L2 regularization are used to prevent model overfitting, SENet attention mechanism is used to improve the model's ability to extract features. At the same time, transfer learning is used to reduce training time and training parameters. Experimental results show that the overall accuracy of woody fruit plant leaf recognition based on this model can reach 85.90%. Compared with the classic ResNet network, the accuracy is increased by 1.20%, and the model parameters are reduced by 98.14%. Therefore, the model proposed in this paper provides a better solution for the identification of leaf diseases of woody fruit plants and has a higher accuracy rate.

## Introduction

Leaf disease of fruit is particularly important, serious leaf disease not only leads to leaf drying off, affecting the late development of fruit and the formation of buds, but also causes the weakening of the tree, bringing about the aggravation of other diseases^1–3^. Fruit leaf disease in addition to causing the decline in the quantity and quality of the fruit itself, in the case of prevention and control is not timely, resulting in other plants infected easily, causing serious economic losses, and it has a huge impact on the environment. In addition, all kinds of fruits are indispensable energy food for human life. Therefore, timely diagnosis and treatment of woody fruit plant leaf disease is an important measure to avoid loss^4–6^. However, due to the variety of plant diseases, it is easy to misjudge by artificial eye observation alone, which leads to woody fruit plant leaf disease can’t be timely control, resulting in food and economic losses^7,8^.

With the rapid development of mobile network and computer technology, some scholars have chosen to use computer vision technology as a method of detecting and identifying plant diseases, and proposed plant leaf disease identification models based on different algorithms. Kaur et al.^9^ extracted the color coherence vector (CCV) and gray level co-occurrence matrix of infected leaves, as well as the combined features of shapes, so as to carry out SVM classification, and the final accuracy could reach 85.65%. Zhang et al.^10^ studied apple leaf disease identification, and took five statistical parameters with hue H in the diseased spot image of each apple leaf as its color features, and then combined them with shape and texture features in the spatial domain, and then use genetic algorithm to do feature selection. After SVM classification, the accuracy rate of 93% is obtained. Although the traditional leaf disease recognition method is relatively effective, the technical threshold is relatively high due to the cumbersome image preprocessing and feature extraction operations.

In recent years, in order to further improve the recognition accuracy of leaf diseases and simplify the experimental process, CNN has gradually been introduced, and it has shown good performance in many recognition tasks^11–15^. Kawasaki et al.^16^ proposed a new plant disease detection system based on Convolutional Neural Network (CNN), which achieved 94.9% accuracy under a fourfold cross-validation strategy. Mohanty et al.^17^ trained a deep convolutional neural network to identify 14 crops and 26 diseases. The data set is 54,306 diseased and healthy plant leaf images collected under controlled conditions. The trained model achieves 99.35% accuracy on the test set. Ramcharan et al.^18^ used transfer learning to train a deep convolutional neural network, which is used to identify three diseases and two types of pests, namely: brown spot (BLS), red mite (MRD), green Mite (GMD), cassava mosaic disease (CMD), cassava brown streak disease (CBSD). The overall accuracy of the best model is 93%. Zhang et al.^19^ proposed an improved GoogLeNet and Cifar10 model based on deep learning for corn leaf disease recognition. By adjusting the parameters, two improved models were obtained, which were used to train and test the images of 9 types of corn leaves (there are 8 types of diseased corn leaves and 1 type of healthy leaves). The improved method improves the recognition accuracy of corn leaf disease, reduces the number of convergence iterations, and effectively improves the training and recognition efficiency of the model. The average recognition accuracy of the GoogLeNet model is 98.9%, and the average recognition accuracy of the Cifar10 model is 98.8%. Selvaraj et al.^20^ created a data set containing samples with classification labels for 1 type of health and 17 types of diseases of banana plants from some hot spots in Africa and southern India. They retrained the ResNet50, InceptionV2 and MobileNetV1 convolutional neural network architectures using transfer learning methods, and obtained 90% accuracy after experiments, and the experiments showed that the models based on ResNet50 and InceptionV2 performed better than MobileNetV1. Uğuz et al.^21^ used the transfer learning method on the VGG16 and VGG19 architectures and the CNN architecture proposed by themselves, and at the same time expanded the data set, and the recognition accuracy rate of up to 95% can be obtained. Although the CNN model has greatly improved the recognition accuracy compared to traditional machine learning methods, there are still many problems. For example, in order to improve the accuracy of the recognition, the network model layer number will be more, so the training parameters will increase, which will lead to the extension of the training time. These problems will affect our application of the model in actual application scenarios.

Based on the study and summary of the above research methods, this paper proposes a method based on transfer learning to identify the leaf diseases of woody fruit plants. This method greatly reduces the training parameters and saves training time. In this paper, the structure and knowledge of the pre-trained ResNet101 model on the large image dataset of ImageNet are transferred to the field of leaf disease recognition of woody fruit plants, and this paper fine-tune the network, delete the top structure, and then add a global average pooling layer, fully connected layer and Softmax, at the same time, training the last layer parameters of the network convolutional layer. Based on the data set used in this article, the proposed model can reduce the training parameters of the original ResNet101 model by 41,759,168, which greatly reduces the training time and improves the recognition accuracy.

## Material and methods

### Data set

The dataset working on this article is from AI Challenger 2018, which includes 10 plants with a total of 27 diseases. By species—pest species—severity, there are 61 categories. The work of this paper focuses on the identification of diseases of woody fruit plant leaves, so select the diseased leaves of woody fruit plants from the data set. There are 25 types of woody fruit plant leaf disease pictures in the data set, including healthy leaves and diseased leaves.

**Figure 1:**
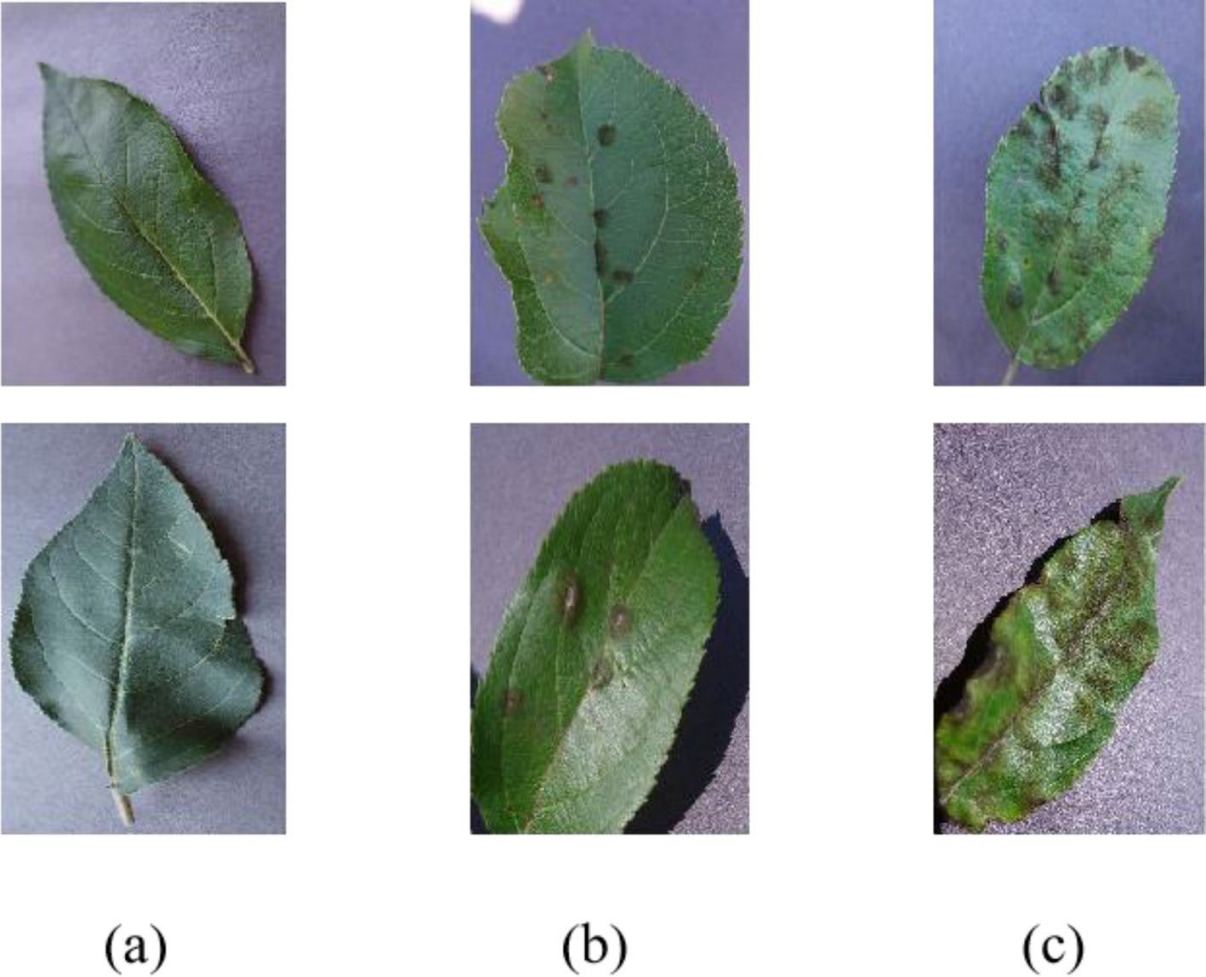
Partial data set.

In this work, we used a total of 14,433 image data sets, which were divided into two subsets, of which 12,620 images were used for model training, and the other 1813 images were used for model testing. Figure 1 shows part of the data set: healthy apple leaves (a); apple leaves with scab and moderate disease (b); apple leaves with scab and severe disease(c).

Considering the small data set of this study, literature^22^ was referred to prove its feasibility. Chen et al. used the VGG model to train on ImageNet, and then transferred the parameters of the model to their own network for training, and completed the identification of 8 maize and rice leaf diseases. The data set they used included 500 rice images and 466 maize images.

### Principles and structure of ResNet

**Figure 2:**
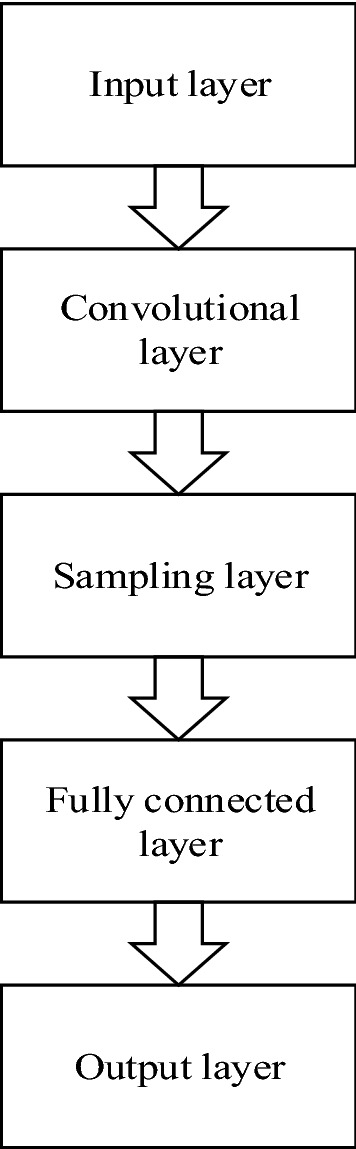
The structure of the convolutional neural network.

Convolutional neural network uses a special network structure^23^, this network structure can speed up the training of multiple hidden layers in the deep layer, which is very suitable for image recognition. Now, convolutional neural networks have been widely used in the field of computer vision, such as image recognition, object detection, semantic segmentation, traffic control, flow forecast and so on^24–26^. Compared with other image recognition algorithms, Convolutional Neural Network (CNN) has relatively less data preprocessing. In general, a common CNN is composed of an input layer, an output layer and multiple hidden layers. The hidden layer of a CNN is usually composed of multiple convolutional layers, multiple sampling layers and fully connected layers. By superimposing these layers, a complete convolutional neural network is formed, and its structure is shown in Fig. 2. Among them, the convolutional layer realizes dimensionality reduction and feature extraction through local perception and parameter sharing. The pooling layer can further reduce the number of parameters in the network and reduce the complexity of the model. The more common pooling operations include maximum pooling, average pooling and L2 pooling. The fully connected layer is mainly used for classification. The output layer is mainly used to output classification results.

ResNet (Residual Neural Network) was proposed by four Chinese people such as Kaiming He of Microsoft Research and others^27^. They successfully trained a 152-layer neural network by using ResNet Unit and won the championship in the ILSVRC2015 competition. In the top 5, the error rate is 3.57%, and the number of parameters is lower than VGGNet, so the recognition effect is very outstanding.

**Figure 3:**
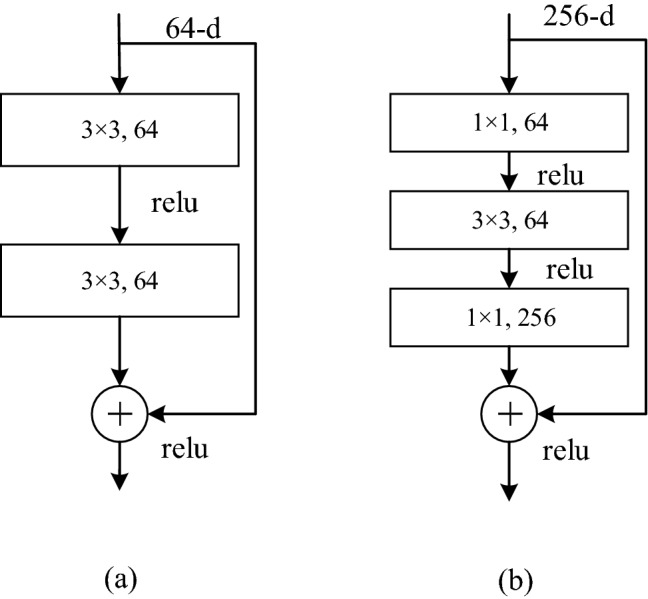
Two residual blocks of ResNet.

Two residual modules are used in the ResNet network structure, one is two 3 × 3 convolutional networks connected in series as a residual module, and the other is that 3 convolutional networks of 1 × 1, 3 × 3, 1 × 1 are serially connected together as a residual module. These are shown in (a) and (b) in Fig. 3 respectively.

**Figure 4:**
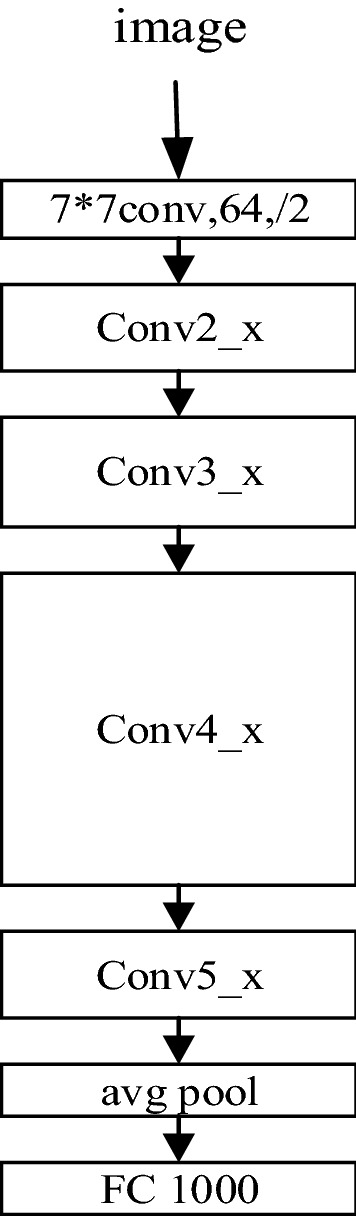
Schematic diagram of ResNet structure.

Among them, the Resnet101 network begins with a convolution layer, followed by four sets of modules consisting of residual blocks, which use the residual blocks shown in Fig. 3b, each group of modules using 3, 4, 23, 3 residual blocks with the same number of output channels. Each module doubles the number of channels for the previous module in the first residual block and Height and width halved. Finally, the output classification layer is connected. Figure 4 shows a diagram of the ResNet structure.

### Transfer learning principle and ResNet improvement scheme

#### Transfer learning principle

In practical applications, training woody fruit plant leaf disease recognition model network requires large-scale data sets, and these data sets are difficult to obtain and label, which is also a common problem in some specific fields (such as: agriculture, medicine). And as the model becomes more and more complex, the number of required parameters and the amount of training data will also increase. Therefore, deep networks generally face problems such as insufficient generalization capabilities for new problems and lack of massive data required for certain specific problems. With the help of transfer learning technology, the pre-trained network model learned from other large data sets (such as ImageNet), after perfection and modification, can be reused in related fields. This method can alleviate the over-fitting problem caused by insufficient data in a large extent. At the same time, transfer learning can solve the problem of small data. When the data source of a particular domain is insufficient, but its data source is associated with a certain domain, the model built in this domain can be migrated and reused. At present, networks that have been trained on large-scale annotated data sets (such as ImageNet, which contains 15 million images) include Alexnet, VGG16, VGG19, Googlenet, etc.^28–31^.

The essence of transfer learning is to transfer and reuse knowledge in other fields. In neural network transfer learning, there are two common strategies: feature extraction and fine-tuning. Feature extraction is to add or modify a simple classifier after the pre-trained network structure, and use the pre-trained network on the source learning task as the feature extractor for another target task. Fine-tuning is to use a pre-trained network to initialize the network model, and use new data to train part or the entire network.

Because of the small number of datasets used in this paper, training convolutional neural networks from scratch takes a long time, and insufficient data can easily lead to overfitted problems, which can seriously affect the performance of the model. Therefore, we chose to use transfer learning to classify and identify woody fruit plant leaf diseases. In this paper, ResNet101 is fine-tuned to fit our data set, which greatly reduces training time and training parameters.

#### Improved convolution neural network based on ResNet101

The classic ResNet solves the degradation problem of the network model, which allows the depth of the neural network to increase without gradient disappearance or gradient explosion. However, when the data set is small, there will still be overfitting. In addition, as the network depth increases, the training time also increases.

**Figure 5:**
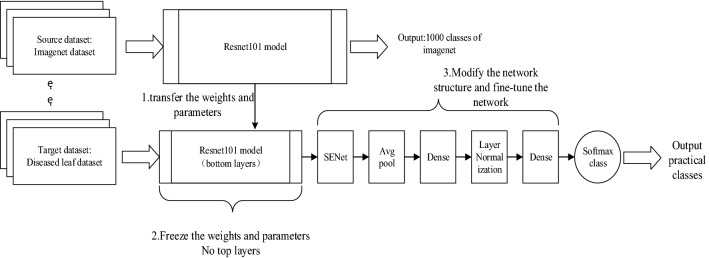
ResNet101 network transfer learning process.

In order to solve these problems, this paper uses the transfer learning method to train the network and adjust the model structure to improve the ResNet101 model. First, the original top structure is removed, attention mechanism is introduced, and we added SENet to improve the capability of feature extraction from the network. Next, a global average pooling layer is added to reduce parameters, and two fully connected layers are added. In the process of model representation capability migration, the full connection layer acts as a firewall to maintain greater model robustness, so as to ensure the migration of model representation capability. In addition, layer normalization is added between the two fully connected layers to further improve the rate of convergence. Finally, a Softmax classifier is used to classify the output. In this paper, we also use Dropout and L2 regularization to prevent overfitting. In order to avoid long-term training, the ResNet101 network weights are pre-trained on ImageNet. In addition, this paper uses the adaptive moment estimation (Adam) method to speed up the convergence of the network. The network transfer learning process diagram is shown in Fig. 5.

(1) SENet

The SE module is the addition of the attention mechanism on the channel. There are two main steps: the squeeze and the excitation. The squeeze operation is the space features on one channel are encoded as global features. Then the excitation is used in the global features. The relationship between channels is learned and the weight of different channels is obtained. The model can focus on the channel features with a large amount of information as much as possible, and suppress the channel features with a small amount of information by this means. Therefore, the network can better learn important features, improve the ability of network to extract features, and improve the sensitivity of the model to channel features. In Fig. 5, SENet is placed after the convolutional layer of ResNet101, mainly to further weight division of the output features of the whole convolutional layer after transfer learning, and to increase the network's attention to effective features.

(2) Global average pooling

In the common convolutional neural network, after the convolutional layer extracts the features of the image, it connects the fully connected layer, and finally connects the activation function for classification. The fully connected layer first expands the convolutional layers into vectors, and then classifies each feature map. The global average pooling layer combines the above two processes of the fully connected layer into one, giving meaning to each channel, so that the entire network can be regularized in structure and effectively and prevent over-fitting. At the same time, the global average pooling layer strengthens the consistency of the feature map and the category, making the convolution structure simpler and more stable.

(3) Dropout and L2 regularization

Dropout can randomly disconnect the neural network. Its meaning is to reduce the number of network model parameters actually involved in calculation in each training, which can prevent overfitting to a certain extent. But in the test, dropout will restore all connections, the purpose is to ensure that the model can get the best performance during the test. In the convolutional neural network, as the dropout layer increases, the actual capacity of the network model during training decreases and the generalization ability increases.

L2 regularization is a regularization calculation method that uses the L2 norm as a sparsity penalty item. It penalizes the square of all parameters in the objective function, and severely penalizes the weight vector of large values, so that the network can use all input features instead of relying on some small features. The use of L2 regularization can effectively prevent network overfitting.

(4) Layer normalization (LN)

As the number of layers of a neural network deepens, small changes in the underlying network are amplified by accumulation. Therefore, the distribution change of training data in the training process has a great influence on the network training speed. In order to solve the problem caused by unstable data distribution of some layers in the training process, the layer normalization method is introduced in this paper.

LN is to do data normalization for all neurons in a layer network. Different from batch normalization, it is a horizontal normalization. LN comprehensively considers the input of all dimensions of a layer network, then calculates the average input value and input variance of the layer, and finally uses the same normalized operation to transform the input of each dimension. It is not affected by batch size, and does not need to open up space to store the mean value and variance of each node, which saves extra storage space. In addition, the use of LN makes the distribution of input data relatively stable, which can accelerate the model training speed and reduce the difficulty of model optimization.

(5) Adam

Adam combines the first-order momentum of SGD and the second-order momentum of RMSProp, which is an extension of the stochastic gradient descent algorithm. The stochastic gradient descent algorithm always maintains a single learning rate for learning and training, and updates the weights. The Adam algorithm calculates the exponential moving average of the gradient and the squared gradient, and uses two parameters to control the attenuation of these values. Therefore, Adam can update the network weights according to the iteration of the training data, which can achieve network convergence faster and improve the training effect. In addition, this method can also effectively solve the problems of other optimizers, such as: the parameter update variance is too large, the convergence speed is too slow, the gradient is sparse or the gradient is very noisy, and the memory requirement is large, etc.

## Results and discussion

In this paper, the Inter Core i5 9400F GPU is used to complete the work, and the model is established through Keras deep learning framework.

### Model performance comparison

**Figure 6:**
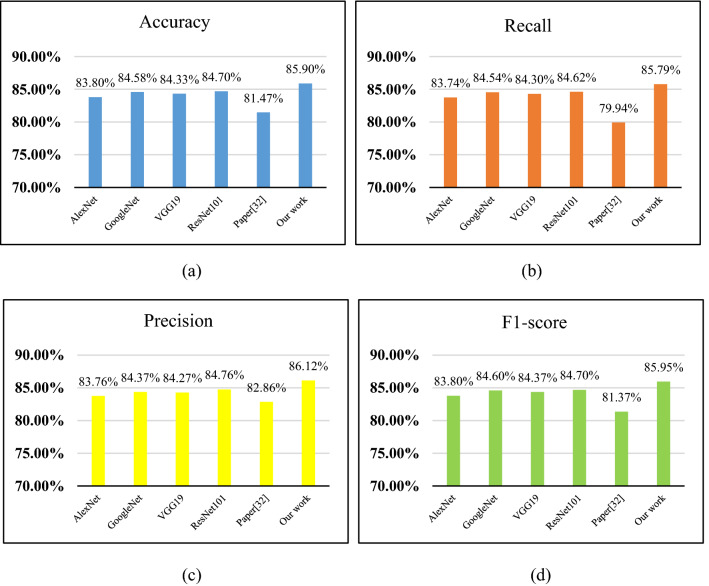
Accuracy comparison of different models.

In order to evaluate the performance of the improved ResNet101 model, we compare the experimental results with the recognition results of four classic convolutional neural networks. In addition, the leaf disease recognition network structure proposed by Yan et al.^32^ is also used for comparison. Performance evaluation indexes of each model are shown in Fig. 6, in which different model recognition accuracy is shown in Fig. 6a. Figure 6b shown the recall rate histogram of the five networks. Figure 6c shown the precision histogram of the five networks, and Fig. 6d is the F1-score histogram of the five networks.

It can be found in the Fig. 6a that the accuracy of AlexNet is 83.80%, the accuracy of VGG19 is 84.58%, the recognition accuracy of GoogleNet can reach 84.33%, ResNet101 can reach 84.70%, and the recognition accuracy of paper^32^ can reach 81.47%. By comparison, it is found that the network identification accuracy proposed in this paper is 4.43% higher than that proposed in paper^32^. In this study, the network adopts the pre-training network model and fine-tunes the network to optimize the entire network structure, and so as to improve the recognition accuracy. Therefore, the highest accuracy, 85.90%, has been achieved in the identification of woody fruit plant leaf diseases in this paper, which can also prove the effectiveness of the model proposed in this study. Compared with the other four models, whether it is accuracy, recall rate or F1-score, the model proposed in this paper has obtained high scores.

As the experiments compared in Fig. 6 trained from scratch, their performance will also be affected if the data set is not large enough. Therefore, this study also uses the SVM method for comparative experiments, linear kernel was used to train the SVM model, and HOG feature extractor was used to extract features. And the results show that the accuracy of SVM obtained on the data set in this study is 80.13%. It can be seen from the results that the method used in this study has more advantages in the identification of leaf diseases of woody fruit plants.

**Figure 7:**
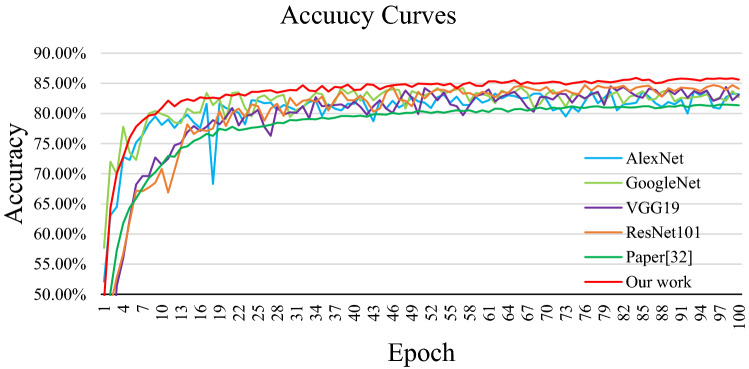
The accuracy curves of each model.

**Figure 8:**
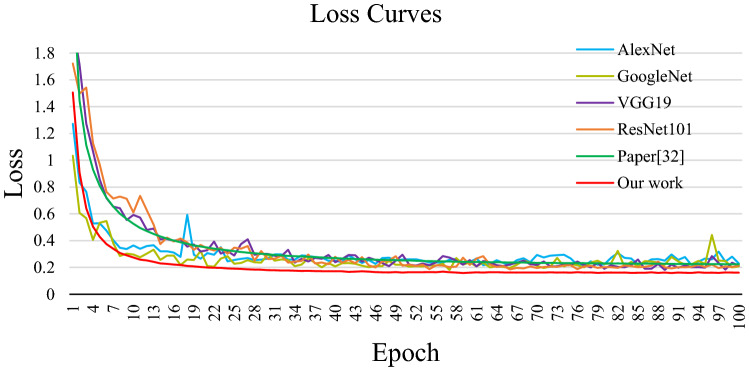
The loss curves of each model.

The loss curves of each model are shown in Fig. 7, and the accuracy curves are shown in Fig. 8. In this study, the loss value is calculated by the cross-entropy loss function and the adaptive moment estimation (Adam) is used as the optimizer. They can improve the convergence speed of the network and improve the training effect. As we can see from the experimental results in Figs. 7 and 8, the convergence speed of the proposed model is faster than the other four CNN models, and the accuracy is also higher than other networks, and the curves’ fluctuation is relatively small. It can also be seen from the Fig. 7 that although the accuracy has been improved, the improvement is small. This is because the data set in this paper is unbalanced, which reduces the improvement effect of the network on the accuracy. For example, some categories have more than 1800 images, while others have as few as 40. In short, experiments show that the proposed network model used in this paper is feasible.

### Training parameters and time

Table 1 shows the number of parameters of each model. It can be seen from the table that the VGG19 model has the most parameters, and the ResNet101 transfer learning model used in this paper has the least. Compared with the original ResNet101 model, our improved model reduces 41,759,168 training parameters. Although the training parameters of the model proposed in this paper are the least, the training time of each round is slightly longer than that of AlexNet and GooleNet, because the depth of this network model is relatively large. The convolutional neural network proposed in this paper has the advantage of fewer training parameters than AlexNet, GoogleNet, VGG19 and ResNet101. In addition, under the condition of minimizing training parameters, the training accuracy and related performance are improved, and the training time is greatly shortened, which is also one of the advantages of the model proposed in this paper.

**Table 1:** Test training parameters of each network.

Model	Parameter	Time(s)/epoch
AlexNet	14,632,665	44.3
GoogleNet	10,380,155	47.3
VGG19	75,654,233	147.4
ResNet101	42,551,385	156.5
Paper^23^	13,849	60.5
Our work	792,217	49.8

## Conclusions

In this study, five convolutional neural network models, namely AlexNet, GoogleNet, VGG19, ResNet101 and the improved ResNet101 model, were used to classify and identify 25 types of disease images with inconsistent disease levels from six different woody plants. In the network training process, we used pre-training models to reduce training parameters and training time, and optimized the network structure, thereby improving network performance, speeding up network convergence, and preventing overfitting. This method has high accuracy, precision, recall rate and F1-score in the identification of leaf diseases of woody fruit plants. Experiments have proved that the improved ResNet101 convolutional neural network model has better indicators than other models.

In future work, we will make improvements in the following two aspects: (1) Collect more disease pictures to enrich the data set, so as to train better models, (2) Try to apply the algorithm of this paper to the hardware platform, and real-time detection of relevant pictures.

## References

[1] Zhang Kaixing Lu, Gaolong JH, Xiuyan Z, Xianxi L (2019). Recognition of corn leaf diseases based on image processing and BP neural network. Chin. J. Agric. Mach. Chem..

[2] Xiaoqing G, Taojie F, Xin S (2019). Image recognition of tomato leaf diseases based on improved Multi-Scale AlexNet. Trans. Chin. Soc. Agric. Eng..

[3] Wenxia B, Jian Z, Dongyan Z, Dong L (2018). Identification of wheat leaf diseases based on ellipse metric learning. Trans. Chin. Soc. Agric. Mach..

[4] Zhiyun X, Hong L (2017). Adaptive feature fusion and rapid recognition of potato typical disease images. Trans. Chin. Soc. Agric. Mach..

[5] Ebrahimi MA, Khoshtaghaza MH, Minaei S (2017). Vision-based pest detection based on SVM classification method. Comput. Electron. Agric..

[6] García, J., Pope, C. & Altimiras, F. A distributed-means segmentation algorithm applied to lobesia botrana recognition. *Complexity* **2017** (2017).

[7] Singh V, Misra AK (2017). Detection of plant leaf diseases using image segmentation and soft computing techniques. Inf. Process. Agric..

[8] Hopkins GW, Freckleton RP (2002). Declines in the numbers of amateur and professional taxonomists: Implications for conservation. Anim. Conserv..

[9] Kaur S, Pandey S, Goel S (2018). Semi-automatic leaf disease detection and classification system for soybean culture. IET Image Proc..

[10] Zhang C, Zhang S, Yang J (2017). Apple leaf disease identification using genetic algorithm and correlation based feature selection method. Int. J. Agric. Biol. Eng..

[11] Hang J, Zhang D, Chen P (2019). Classification of plant leaf diseases based on improved convolutional neural network. Sensors.

[12] Sladojevic, S., Arsenovic, M., Anderla, A. *et al*. Deep neural networks based recognition of plant diseases by leaf image classification. *Comput. Intell. Neurosci*. **2016** (2016). 10.1155/2016/3289801 PMC4934169 27418923

[13] Ren, S., He, K., Girshick, R. *et al*. Faster r-cnn: Towards real-time object detection with region proposal networks. arXiv preprint arXiv:1506.01497 (2015). 10.1109/TPAMI.2016.2577031 27295650

[14] Mahlein AK (2016). Plant disease detection by imaging sensors–parallels and specific demands for precision agriculture and plant phenotyping. Plant Dis..

[15] Fuentes A, Yoon S, Kim SC (2017). A robust deep-learning-based detector for real-time tomato plant diseases and pests recognition. Sensors.

[16] Kawasaki, Y., Uga, H., Kagiwada, S. *et al*. Basic study of automated diagnosis of viral plant diseases using convolutional neural networks. International Symposium on Visual Computing 638–645 (Springer, 2015).

[17] Mohanty SP, Hughes DP, Salathé M (2016). Using deep learning for image-based plant disease detection. Front. Plant Sci..

[18] Ramcharan A, Baranowski K, McCloskey P (1852). Deep learning for image-based cassava disease detection. Front. Plant Sci..

[19] Zhang X, Qiao Y, Meng F (2018). Identification of maize leaf diseases using improved deep convolutional neural networks. IEEE Access.

[20] Selvaraj MG, Vergara A, Ruiz H (2019). AI-powered banana diseases and pest detection. Plant Methods.

[21] Uğuz, S. & Uysal, N. Classification of olive leaf diseases using deep convolutional neural networks. *Neural Comput. Appl.,* 1–17 (2020).

[22] Chen J, Chen J, Zhang D (2020). Using deep transfer learning for image-based plant disease identification. Comput. Electron. Agric..

[23] Lawrence S, Giles CL, Tsoi AC (1997). Face recognition: A convolutional neural-network approach. IEEE Trans. Neural Netw..

[24] Jindal A, Aujla GS, Kumar N (2018). SeDaTiVe: SDN-enabled deep learning architecture for network traffic control in vehicular cyber-physical systems. IEEE Netw..

[25] Garg S, Kaur K, Kumar N (2019). Hybrid deep-learning-based anomaly detection scheme for suspicious flow detection in SDN: A social multimedia perspective. IEEE Trans. Multimedia.

[26] Miglani A, Kumar N (2019). Deep learning models for traffic flow prediction in autonomous vehicles: A review, solutions, and challenges. Veh. Commun..

[27] He, K., Zhang, X., Ren, S. *et al*. Deep residual learning for image recognition. Proceedings of the IEEE Conference on Computer Vision and Pattern Recognition 770–778 (2016).

[28] Krizhevsky A, Sutskever I, Hinton GE (2017). Imagenet classification with deep convolutional neural networks. Commun. ACM.

[29] Simonyan, K. & Zisserman, A. Very deep convolutional networks for large-scale image recognition. arXiv preprint arXiv:1409.1556 (2014).

[30] Szegedy, C., Liu, W., Jia, Y. *et al*. Going deeper with convolutions. Proceedings of the IEEE Conference on Computer Vision and Pattern Recognition 1–9 (2015).

[31] Khan A, Sohail A, Zahoora U (2020). A survey of the recent architectures of deep convolutional neural networks. Artif. Intell. Rev..

[32] Yan Q, Yang B, Wang W (2020). Apple leaf diseases recognition based on an improved convolutional neural network. Sensors.

